# Kynurenic acid as the neglected ingredient of commercial baby formulas

**DOI:** 10.1038/s41598-019-42646-4

**Published:** 2019-04-15

**Authors:** Pawel Milart, Piotr Paluszkiewicz, Piotr Dobrowolski, Ewa Tomaszewska, Katarzyna Smolinska, Iwona Debinska, Kinga Gawel, Katarzyna Walczak, Jerzy Bednarski, Monika Turska, Michal Raban, Tomasz Kocki, Waldemar A. Turski

**Affiliations:** 1 3rd Department of Gynecology, Medical University of Lublin, Jaczewskiego 8, PL-20090 Lublin, Poland; 2 Department of General, Oncological and Metabolic Surgery Institute of Haematology and Transfusion Medicine, Indiry Gandhi 14, PL-02776 Warsaw, Poland; 3 Department of Surgery and Surgical Nursing, Medical University of Lublin, Szkolna 18, PL-20124 Lublin, Poland; 4 Department of Comparative Anatomy and Anthropology, Maria Curie-Sklodowska University, Akademicka 19, PL-20033 Lublin, Poland; 5 Department of Animal Physiology, University of Life Sciences in Lublin, Akademicka 12, PL-20950 Lublin, Poland; 6 Department of Experimental and Clinical Pharmacology, Medical University of Lublin, Jaczewskiego 8b, PL-20090 Lublin, Poland; 7 Department of Pharmacology, Medical University of Lublin, Chodzki 4a, PL-20093 Lublin, Poland; 8 Chair of Human Anatomy (Department of Normal Anatomy), Medical University of Lublin, Jaczewskiego 4, PL-20090 Lublin, Poland; 9 2nd Chair and Department of General and Gastrointestinal Surgery and Surgical Oncology of the Alimentary Tract, Medical University of Lublin, Staszica 16, PL-20081 Lublin, Poland

## Abstract

The global increase in resorting to artificial nutritional formulas replacing breastfeeding has been identified among the complex causes of the obesity epidemic in infants and children. One of the factors recently recognized to influence metabolism and weight gain is kynurenic acid (KYNA), an agonist of G protein-coupled receptor (GPR35). Therefore the aim of the study was to determine the concentration of KYNA in artificial nutritional formulas in comparison with its level in human breast milk and to evaluate developmental changes in rats exposed to KYNA enriched diet during the time of breastfeeding. KYNA levels were measured in milk samples from 25 heathy breast-feeding women during the first six months after labor and were compared with 21 time-adjusted nutritional formulas. Animal experiments were performed on male Wistar rats. KYNA was administered in drinking water. The content of KYNA in human milk increases more than 13 times during the time of breastfeeding while its level is significantly lower in artificial formulas. KYNA was detected in breast milk of rats and it was found that the supplementation of rat maternal diet with KYNA in drinking water results in its increase in maternal milk. By means of the immunoblotting technique, GPR35 was evidenced in the mucosa of the jejunum of 1-day-old rats and distinct morphological changes in the jejunum of 21-day-old rats fed by mothers exposed to water supplemented with KYNA were found. A significant reduction of body weight gain of rats postnatally exposed to KYNA supplementation without changes in total body surface and bone mineral density was observed. The rat offspring fed with breast milk with artificially enhanced KYNA content demonstrated a lower mass gain during the first 21 days of life, which indicates that KYNA may act as an anti-obesogen. Further studies are, therefore, warranted to investigate the mechanisms regulating KYNA secretion via breast milk, as well as the influence of breast milk KYNA on mass gain. In the context of lifelong obesity observed worldwide in children fed artificially, our results imply that insufficient amount of KYNA in baby formulas could be considered as one of the factors associated with increased mass gain.

## Introduction

The global phenomenon of overweight and obesity and its negative impact on health and quality of life have attracted a growing interest in mechanisms and processes responsible for the emergence of these comorbidities. Hence, the beginning of the millennium witnessed the onset of the concept of *obesogens, i.e*., environmental chemicals affecting obesity. The concept further evolved to comprise both chemical and non-chemical stressors^1^. Moreover, endocrine disrupting chemicals, which influence the adipose tissue, exhibit strong obesogenic features^2^. Importantly, current epidemiological studies alarmingly indicate artificial formulas as a risk factor for obesity at later stages of child development^3,4^ The superiority of breastfeeding over usage of artificial formulas in terms of health benefits is presently beyond dispute. Consequently, as we have recently remarked^5^, it is justified to explore whether obesogens are overrepresented and/or whether anti-obesogens are deficient in artificial formulas and thus conducive to obesity.

Quite recently, a study carried out by Agudelo *et al*., on mice with genetically deleted GPR35 showed an increased weight gain and impaired glucose tolerance. Furthermore, the authors demonstrated that KYNA caused increased energy expenditure and reduced body weight due to activation of GPR35^6^. Interestingly, the highest expression level of GPR35 was detected in the gastrointestinal tract in humans and it was also evident that KYNA concentration in jejunum is sufficient enough to activate these receptors^7^.

KYNA is a metabolite of tryptophan produced on the kynurenine metabolic pathway. It is constantly present in tissues and body fluids (see review^8^). It can be found in food, e.g. milk (see review^8^). However, its action upon the development of the digestive tract has never been investigated before. Currently, the only indication for such a possibility is the presence of KYNA in human amniotic fluid^9^ that fills fetal digestive lumen, and in human breast milk^10^. Nevertheless, other sources such as production of KYNA by intestinal mucous cells or microbiome should be considered^11^.

## Material and Methods

### Humans

Breast milk was obtained from 25 heathy breast-feeding women during the first six months after labor. The study protocol was approved by the Bioethics Committee of the Medical University of Lublin, Poland (KE-0254/168/2009). Written consent was obtained from each woman under the study. The milk samples were collected 6 times: on 3^rd^ and 7^th^ day, 2^nd^ week and 1^st^, 3^rd^, 4^th^, 5^th^ and 6^th^ month after the delivery. The women were instructed how to collect their breast milk. The samples of human breast hindmilk, after the first breastfeeding of the day, were collected by means of breast pumps in the amount of 5 mL to plastic containers and stored in a fridge for no longer than three hours, then, in human tissue transport boxes were delivered to the lab, and transferred to glass sterile probes in the volume of 1 mL. Perchloric acid (2 M) was added to the milk samples in the ratio of 1:5 and the samples were centrifuged at 6,000 g for 15 minutes. Supernatant was stored in −20 °C prior to the laboratory analysis. All the analyses were performed in accordance with the relevant guidelines and regulations.

### Animals

Experiments were performed on male and female Wistar rats. The animals were kept in standard laboratory conditions with food and water available *ad libitum*. Objects forming an enriched environment that increase physical activity of animals were not present in the housing cages. All experimental protocols were approved by Local Ethics Committee for Animal Experiments in Lublin (31/2009; 28/2014; 37/2017). The rats were weighed on day 2, 4, 8, 12, 16 and 20 after birth. KYNA was dissolved in drinking water for rats in the concentration of 250 mg/L (approx. 25 mg/kg b. w./day).

### Chemicals

Kynurenic acid (KYNA) was obtained from Sigma-Aldrich. High performance liquid chromatography (HPLC) reagents were purchased from J.T. Baker Chemicals and from Sigma-Aldrich. All the other chemicals used were of the highest commercially available purity.

### Infant nutritional formulas

21 standard feeding formulas designed for alternative feeding of human infants were commercially purchased (for brand names and suppliers see Table 3). Three packages of three different series of batches of the same product were used for analysis. The samples of milk formulas were prepared according the producers’ recipes. Perchloric acid (2 M) was added to the milk samples in the ratio of 1:5 and the samples were centrifuged at 6,000 g for 15 minutes. Supernatant was stored in −20 °C prior to the laboratory analysis.

### Kynurenic acid determination

The deproteinized supernatants were applied to the Dowex 50 W^+^ cation-exchange column prewashed with 0.1 N HCl. Subsequently, the column was washed with 1 mL 0.1 N HCl and 1 mL distilled water. Fraction containing KYNA was eluted with 4 mL of distilled water. KYNA content was evaluated by means of HPLC (Hewlett Packard 1046 A; ESA catecholamine HR-80, 3 μm, C18 reverse-phase column, mobile phase: 250 mM zinc acetate, 25 mM sodium acetate, 5% acetonitrile, pH 6.2, flow rate 1.0 ml/min) with a fluorescence detector (excitation 344 nm, emission 398 nm) according to Shibata’s method^12^. The details of method validation of KYNA determination were described by us previously; limit of detection was 0.00002 µg/100 µl, limit of quantitation was 0.00006 µg/100 µl^13^. Authentic KYNA obtained from Sigma was used as an internal standard. A known amount of authentic KYNA was added to the samples before the protein precipitation step. Endogenous KYNA content was calculated against an internal standard.

### Measurement by dual energy x-ray absorptiometry (DEXA)

The animals were scanned using Hologic Discovery W QDR Series DEXA system (Hologic Inc. Bedford, MA, USA). The rats were ventrally positioned and scanned to determine the parameters of body surface [cm^2^] and bone mineral density [g/cm^2^]. The analysis was performed using the small animal mode of the APEX 3.0.1 Software for Windows XP Service Pack 3. The instrument was calibrated at each start.

### Morphometric analysis

Samples of small intestine obtained from each 21-day-old animal were collected and fixed in 4% buffered formaldehyde (pH 7.0) for 12 hours, then dehydrated in graded ethanol solutions, cleared in xylene and embedded in paraffin. Cross sections of 4 µm thick were cut with a microtome (Microm HM 360, Microm, Walldorf, Germany) from every sample of the small intestine. Three methods of staining were used: the Goldner’s trichrome, hematoxylin and eosin and Hoechst plus eosin as described previously^14,15^. Microscopic (two-dimensional) images of each slice were taken using a confocal microscope (AXIOVERT 200 M, Carl Zeiss, Jena, Germany) equipped with a color digital camera (AxioCam HRc, Carl Zeiss, Jena, Germany). The analysis of collected images was performed using graphic analysis software ImageJ 1.52 (National Institute of Health USA, http://rsb.info.nih.gov/ij/index.html). The following parameters according to small intestine histomorphometry were analyzed: mucosa, submucosa, and myenteron thickness, crypt depth and width, the number of crypts, villus height and width, the number of villi per millimeter of mucosa, and the number of enterocytes per 100 µm of villus epithelium, villus epithelium thickness, the number of mitoses calculated per millimeter of crypt epithelium; apoptotic cells were counted per square millimeter of tissue and small intestinal absorptive surface as well as the villi length to crypt depth ratio were calculated.

### Western blot analysis

Samples of 1-day-old rat small intestine (3.5 cm long) previously frozen at −80 °C were lysed in modified RIPA buffer (150 mM NaCl, 50 mM Tris-HCl pH 7.4, 1 mM EDTA, 1% Triton X-100, 1% sodium deoxycholate, 0.1% SDS, 1 mM PMSF, protease inhibitor mixture), sonicated and centrifuged at 14,000 g for 10 min. Protein expression of GPR35 and β-actin was assessed by western blot previously described^16^ (primary antibodies: GPR35 1:1,000, Thermo Fisher Scientific, Rockford, USA; β-actin 1:2,000, Cell Signaling Technology, Danvers, USA; HRP- conjugated secondary antibodies 1:2,000, Cell Signaling Technology).

### Statistics

The data are presented as a mean ± standard error of mean (SEM) or median with range (min-max). Statistical analysis was accomplished using one-way ANOVA followed by post hoc Tukey test or t-Student test. A p-value of less than 0.05 was considered significant.

## Results

**Figure 1:**
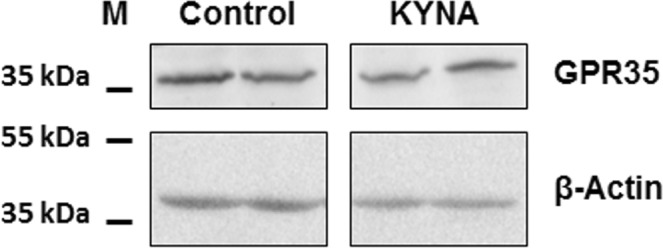
GPR35 in mucosa of jejunum in 1-day-old rat. KYNA (250 mg/L) was administered in drinking water to pregnant rats for the entire gestation period.

**Figure 2:**
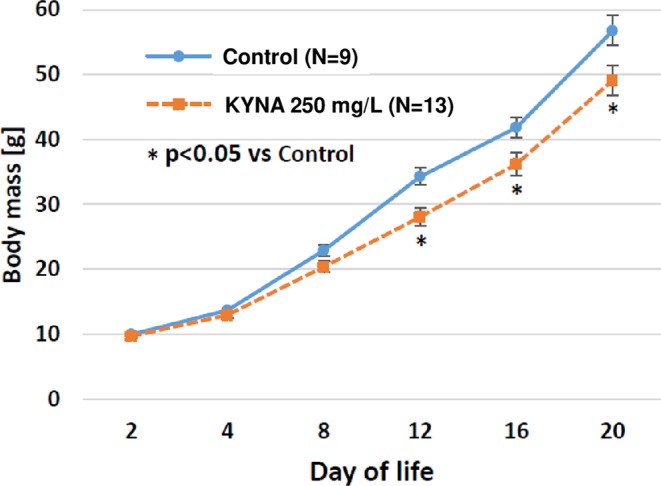
Effect of KYNA on body mass gain in suckling male rats.

It was found that the concentration of KYNA in human milk increased from 3.9 µg/L in the 3^rd^ day to 56.6 µg/L in 6 months after delivery (Table 1). KYNA was found in all the studied artificial baby formulas (Table 2). However, in comparison with human milk with its content naturally changing over time, the concentration of KYNA in artificial formulas was substantially lower (see Table 1 for details). In the study performed in rats, it was discovered that KYNA is present in breast milk of control dams immediately after labor in concentration of 15.9 µg/L (Table 3). The supplementation of the dams’ diet with KYNA in drinking water resulted in an increase in KYNA content in milk harvested from stomachs of 1-day-old rats (Table 3). Western blot analysis revealed that GPR35 was present in the mucosa of the jejunum in 1-day-old rats (Fig. 1). Distinct morphological changes in the jejunum of 21-day-old rats fed by these dams i.e. increase in both intestinal surface absorption and mucosa thickness as well as the enhancement of the number of mitosis in the intestinal crypt were detected (see Table 4 for details). In rats postnatally exposed to KYNA, an attenuation of body mass gain, statistically significant since 12^th^ postnatal day, was observed (Fig. 2). The analysis of body composition of 21-day-old rats indicated that KYNA supplementation affected neither total body surface nor bone mineral density (Table 5).

**Table 1:** Content of kynurenic acid (KYNA) in human breast milk and in artificial nutritional formulas.

Human breast milk		Artificial nutritional formula		
Breastfeeding period	KYNA content [µg/L]	Feeding period [month]	KYNA content [µg/L]	p < versus respective human milk
3 day	3.9 ± 0.6	0–3	5.0 ± 0.7	NS
6 day	10.7 ± 2.3			0.05
14 day	21.1 ± 3.4*			0.001
1 month	37.4 ± 4.5*			0.001
3 month	41.5 ± 5.5*			0.001
4 month	43.1 ± 4.8*	4–5	4.8 ± 1.0	0.001
5 month	47.5 ± 8.9*			0.001
6 month	56.6 ± 8.6*	6–over	7.3 ± 0.9	0.001

Data are presented as mean ± SEM; t-Student test: *p < 0.05 versus 3 day.

**Table 2:** Content of KYNA in studied artificial nutritional formulas.

Brand	Producer	median	minimum	maximum
Bebilon 1 Immuno fortis comfort	Nutricia, Warsaw, Poland	0.33	0.20	0.45
Bebilon AR 1		0.64	0.51	0.77
Bebilon Junior 3		1.18	1.1	1.22
Bebilon Junior 4		1.03	1.01	1.05
Bebilon Pepti 2		0.38	0.33	0.47
Bebilon 1 Immuno fortis		1.08	0.98	1.27
Bebilon 2 Immuno fortis		0.99	0.7	1.4
Bebilon 1 HA Immuno fortis		0.21	0.18	0.28
Bebilon HA 2		0.29	0.28	0.31
Bebilon Comfort 2		0.47	0.3	0.78
Bebilon Pepti 1		0.35	0.31	0.41
Bebilon Nenatal premium		0.85	0.64	1.18
Bebiko 1		0.93	0.83	1.08
Bebiko 2		0.71	0.69	0.73
Bebiko 2 R		0.7	0.63	0.77
Bebiko HA 1		0.25	0.23	0.27
Bebiko HA 2		0.32	0.29	0.36
Bebiko Junior 3		0.65	0.58	0.69
Bebiko Junior 4		0.66	0.65	0.68
Bebilon Nenatal premium		0.74	0.72	0.75
Bebilon Pepti solution		0.33	0.29	0.35
Bebilon 1		0.44	0.39	0.53
Enfamil Premium 1	Mead Johnson Nutrition, Warsaw, Poland	0.67	0.60	0.77
Enfamil Premium 2		0.80	0.72	0.84
Enfamil AR 1		0.89	0.83	0.96
Enfamil AR 2		1.19	0.95	1.54
Enfamil Premium 1		0.51	0.49	0.53
HIPP Bio 1	HIPP, Warsaw, Poland	1.03	0.99	1.08
HIPP Bio 2		1.42	1.38	1.45
HIPP Bio 3		1.52	1.28	1.74
NAN 1	Nestle, Warsaw, Poland	0.25	0.22	0.27
NAN 2		0.54	0.43	0.64
NAN HA 1		0.09	0.07	0.12
NAN HA 2		0.31	0.19	0.40
NAN HA 3		0.28	0.21	0.36
NAN AR		0.37	0.24	0.53
NAN Active 1		0.24	0.20	0.30
NAN Active 2		0.73	0.68	0.78
NAN PRO night B		0.95	0.77	1.05
NAN PRO 3		0.98	0.93	1.03
NAN 2 R		0.71	0.33	1.06
NAN 3 R		1.08	1.08	1.08
NAN PRO 1		0.31	0.29	0.36
NAN PRO HA 1		0.07	0.06	0.07
Nutramigen 1 hypoallergic	Mead Johnson Nutrition, Warsaw, Poland	0.66	0.42	1.07
Nutramigen 2 hypoallergic		0.58	0.43	0.76

**Table 3:** Content of kynurenic acid in breastfeeding rat dam milk^#^.

Group	N	KYNA content [µg/L]
Control	11	15.9 ± 2.4
KYNA 250 mg/L	19	38.1 ± 4.6*

^#^investigated material was collected from the gastric lumen of suckling 1-day-old rats; N – number of subjects, data are presented as a mean ± SEM; * t-Student test: p < 0.05 vs Control.

**Table 4:** Effect of kynurenic acid on histomorphometric parameters of jejunum in 21-day-old male rats.

Group	N	Intestine absorptive surface [µm^2^]	Mucosa thickness [µm]	Total number of villi [/mm^2^]	Total crypt number [/mm^2^]	Number of mitosis in the intestinal crypt
Control	10	6.5 ± 1.2	410.0 ± 13.1	5.5 ± 0.6	18.0 ± 1.5	0.62 ± 0.64
KYNA 250 mg/L	10	7.9 ± 1.4*	454.5 ± 27.4*	5.8 ± 0.3	18.2 ± 3.1	1.68 ± 1.08*

N – number of subject, data are presented as a mean ± SEM; *t-Student test: p < 0.05 vs Control.

**Table 5:** Effect of kynurenic acid on body parameters in 21-day-old male rats.

Group	N	Body mass [g]	Body surface area [cm^2^]	Bone mineral density [g/cm^2^]
Control	16	46.01 ± 0.73	13.21 ± 0.29	0.073 ± 0.001
KYNA 250 mg/L	14	42.89 ± 1.05*	12.55 ± 0.48	0.072 ± 0.001

N – number of subject, data are presented as a mean ± SEM; *t-Student test: p < 0.05 vs Control.

## Discussion

Here we assessed the KYNA concentrations in milk collected from breastfeeding women during the first six months after delivery. Our study indicated that KYNA is present in human milk and its content increased more than 13 times during the studied breastfeeding time.

Recently, O’Rourke *et al*. (2018) studied the content of tryptophan, kynurenine and KYNA in preterm and term expressed breast milk on day 7 and 14 after a normal delivery. They found a higher content of kynurenine and KYNA on day 14 in comparison to day 7 in term group mothers^10^. Our findings are compatible with this result. Furthermore, in the study by O’Rourke *et al*. the total and free tryptophan levels were unchanged. Interestingly, there was no difference in the content of all the three measured compounds in milk collected on day 7 and 14 of lactating mothers who delivered preterm infants^10^. Schröcksnadel *et al*. (2003) reported decreasing tryptophan and increasing kynurenine concentration in blood during pregnancy and increasing content of both compounds postpartum^17^. The concentration of KYNA in blood during pregnancy did not alter significantly^18^. In the rat study we found out that KYNA was present in breast milk of control dams shortly after labor. Moreover, our results showed that supplementation of the dams’ diet with KYNA provided in drinking water resulted in an increase in KYNA content in milk harvested from stomachs of 1-day-old rats. The chosen dose −250 mg/L in drinking water referring to 25 mg/kg b. w./day was the lowest of the doses used in the preliminary experiments resulting in a significant increase in KYNA content in milk without toxic effects as evidenced previously^19,20^.

Previously, we reported the concentration of KYNA in human amniotic fluid at the level of 210 µ/L^9^, and in this study we proved that GPR35 is present in the mucosa of the jejunum in 1-day-old rats. This warrants a hypothesis about an interaction of KYNA ingested from amniotic fluid and subsequently from breast milk, with GPR35, which affects the development of the gastrointestinal tract.

In fact, we indicated distinct morphological changes in the jejunum of 21-day-old rats fed by dams exposed to KYNA supplemented in drinking water. Eventually, an attenuation of body mass gain of rats postnatally exposed to KYNA, compared with controls was observed. On the other hand, the analysis of body composition of 21-day-old rats indicated that KYNA supplementation affected neither total body surface nor bone mineral density. It should also be considered that, apart from milk, small intestine microbiome might be a significant source of KYNA in the breast-fed rat offspring^21^.

Our findings are in accordance with the report by Agudelo *et al*. performed on adult mice. In that study, body weight gain was reduced due to KYNA-dependent activation of GPR35 leading to increased energy expenditure, improved energy metabolism and inflammation, especially in adipose tissue^6^. It still remains to be examined whether similar processes could be induced in early postnatal development.

The molecular mechanism of the action of KYNA exerted through GPR35 is not fully understood. It should be stressed that the knowledge concerning GPR35 is currently still in its infancy. For the first time Wang *et al*. (2006) suggested the signaling function for KYNA through GPR35 activation as recently as in 2006. This group of researchers detected the presence of GPR35 predominantly in the gastrointestinal tract and immune cells and reported calcium mobilization and inositol phosphate production upon KYNA exposure^7^. Shortly afterwards, Guo *et al*. (2008) found the inhibition of N-type calcium channels by activation of GPR35 by KYNA in rat sympathetic neurons^22^. In astrocytes, GPR35 activation resulted in the reduction of forscolin-induced cAMP production and modulation of calcium ion waves produced by exposition of cells to ATP^23^. In 2018, Agudelo *et al*., carefully examined the GPR35-dependent components of KYNA action in the adipose tissue^7^. They proved activation of Ca^2+^, ERK, CREB signaling, stabilization of Pgc-1α1 and induction of downstream genes as well as an increase in Rgs 14 gene expression leading to enhanced β-adrenergic receptor signaling^7^. Recently, Schneditz *et al*. reported that GPR35 interacted with sodium potassium pump and suggested central signaling and metabolic pacesetter function of GPR35 in macrophages and intestinal epithelial cells^24^. At this point, it is clearly premature to unequivocally identify the mechanism(s) responsible for weight gain modulation by KYNA. The role of total energy expenditure affected by KYNA acting on GPR35 receptors seems to be the most likely cause of weight gain control as suggested by Agudelo and colleagues^6^. Although not yet fully understood, other mechanism should also be considered. Due to the fact that β-adrenergic mechanisms are involved in weight gain control^25^, the role of enhancement of this signaling pathway by KYNA should be investigated in depth. Moreover, the recently described strong expression of GPR35 on vagal afferent innervating gastrointestinal mucosa suggests the specific interface connecting microbiome/mucosal and central nervous system^26^, thus allowing the creation of a scenario in which food-derived chemokine, eg. KYNA, acting on GPR35 receptor affects brain centers of appetite and satiety or energy homeostasis^27^. These mechanisms can be also considered as a cause of weight gain reduction in rat offspring fed with KYNA-enriched breast milk. Moreover, there is growing evidence linking inflammation and obesity^28,29^. It has been found that GPR35 expression is substantially high in immune cells^7^. Moreover, activation of this receptor stimulates anti-inflammatory gene expression in adipose tissue^6^. The anti-inflammatory action of KYNA is well established^30,31^. Thus, one cannot exclude that potentially anti-obesic action of KYNA might be associated with its anti-inflammatory mechanisms. Finally, the increase in intestinal absorption area and the lowering of weight gain in KYNA exposed rodents reported here suggest a more complex, multimodality mechanism of weight control connected with activation of various receptors. Noteworthily, KYNA, apart from its influence on GPR35, is a ligand of the aryl hydrocarbon receptor (AhR). It was reported that the diet rich in AhR ligands links to the expression of genes responsible for enterocyte differentiation, the turnover rate and epithelial cells lineage fate^32^. AhR may also be implicated in the occurrence of obesity/adiposity, although currently available data are ambiguous^32^.

Irrespective of what KYNA’s mechanism of action would be, it appears as the first ever defined ingredient of food responsible for weight gain in the postnatal development as revealed in our rat study. To complement this experiment we determined the content of KYNA in selected artificial formulas for infants. Twenty-one of them contained KYNA. Its content, however, in comparison with human milk was, as a rule, much lower. In particular, in artificial nutritional formulas designed for 4–5 month infants it was even 10 times lower. This finding is surprising because the content of protein, carbohydrates and other biologically active food constituents is usually substantially higher in comparison to breast milk. The oversupply of readily absorbed food components in baby formulas is currently considered a risk factor leading to obesity^3,4^. Here, we assume that underrepresentation of KYNA in baby formulas compared to human breast milk may be an unanticipated aspect in the epidemic of obesity in young people.

The results regarding KYNA as an anti-obesogen seem to be a promising suggestion, although some limitation of this study may be indicated. Our study was conducted on the Polish population of breastfeeding mothers. The precise estimation of breast milk intake in rodents was not performed due to permanent sucking observed in offspring. In our experimental paradigm a high-fat diet was not used. The rats had free and unrestricted access to food and water and no objects forming enriched environment that increase physical activity of animals were present in the housing cages. Currently, it is postulated that such conditions lead to obesity as well as premature morbidity and mortality of laboratory animals^33,34^. Therefore, it can be assumed that the parameters of animals belonging to the control group kept under such environmental conditions reflected signs of moderate obesity.

Summing up, the role of KYNA in weight gain control and obesity in children deserves special attention. The influence of KYNA enriched diet on its concentration in breast milk presented in our analysis should be considered in further studies dedicated to the optimization of breastfeeding mothers’ diet. Given that foods rich in KYNA are well identified, the issue should not pose a challenge in its implementation^8,35,36^. The nutritional habits and role of western-diet may be considered in relation to final KYNA concentration in breast milk. Further studies are necessary before artificial formulas with KYNA content replicating this found in human breast milk are introduced. Moreover, our findings indicate that studies on obesity prevention should concern natural micro-compounds capable of counter-acting the obesity programming.
